# A C5a-Immunoglobulin complex in chronic lymphocytic leukemia patients is associated with decreased complement activity

**DOI:** 10.1371/journal.pone.0209024

**Published:** 2019-01-02

**Authors:** Regina Michelis, Tamar Tadmor, Masad Barhoum, Mona Shehadeh, Lev Shvidel, Ariel Aviv, Galia Stemer, Najib Dally, Naomi Rahimi-Levene, Mona Yuklea, Andrei Braester

**Affiliations:** 1 The Institute for Medical Research, Galilee Medical Center, Nahariya, Israel; 2 Hematology Division, Bnai Zion Medical Center, Haifa, Israel; 3 The Ruth and Bruce Rappaport Faculty of Medicine, Technion, Haifa, Israel; 4 Institute of Hematology, Galilee Medical Center, Nahariya, Israel; 5 Faculty of Medicine in the Galilee, Bar Ilan University, Safed, Israel; 6 Biochemistry Laboratory, Galilee Medical Center, Nahariya, Israel; 7 Hematology Institute, Kaplan Medical Center, Rehovot, Israel; 8 Faculty of Medicine, Hebrew University, Jerusalem, Israel; 9 Department of Hematology, Emek Medical Center, Afula, Israel; 10 Department of Hematology, Ziv Medical Center, Zefat, Israel; 11 Hematology Unit, Assaf Harofeh Medical Center, Zerifin, Israel; 12 Sackler Faculty of Medicine, Tel-Aviv University, Tel-Aviv, Israel; 13 Hematology Unit, Meir Medical Center, Kfar Saba, Israel; Cornell University, UNITED STATES

## Abstract

Chronic lymphocytic leukemia (CLL) is the most common adult leukemia in the Western world. The therapeutic approach to CLL includes chemotherapeutic regimens and immunotherapy. Complement-mediated cytotoxicity, which is one of the mechanisms activated by the therapeutic monoclonal antibodies, depends on the availability and activity of the complement (C) system. The aim was to study the structure of circulating C components and evaluate the importance of C5 structural integrity for C activity in CLL patients. Blood samples were collected from 40 naïve CLL patients and 15 normal controls (NC). The Western blot analysis showed abnormal C5 pattern in some CLL patients, while patterns of C3 and C4 were similar in all subjects. Levels of the C activation markers sC5b-9 and C5a were quantified before and after activation via the classical (CP) and alternative (AP) pathways. In patients with abnormal C5, basal levels of sC5b-9 and C5a were increased while activities of the CP and of the CP C5-convertase, the immediate C5-upstream complex, were decreased compared to NC and to patients with normal C5. The data indicate a link between CP activation and apparent C5 alterations in CLL. This provides a potential prognostic tool that may personalize therapy by identifying a sub-group of CLL patients who display an abnormal C5 pattern, high basal levels of sC5b-9 and C5a, and impaired CP activity, and are likely to be less responsive to immunotherapy due to compromised CP activity.

## Introduction

Chronic lymphocytic leukemia (CLL) is the most common adult leukemia in the Western world, accounting for 30% of all leukemias. It is characterized by small, monoclonal B lymphocytes exhibiting mature phenotype that accumulate in peripheral blood, bone marrow, spleen, and secondary lymphoid organs. CLL is diagnosed by presentation of ≥5000 monoclonal B lymphocytes/μl (with membranal κ or λ) in peripheral blood that co-express CD5, CD19, CD20 (weak) and CD23. CLL therapy includes chemotherapy regimens of purine analogues (such as fludarabine) and immunotherapy, which has emerged as a promising treatment option for CLL [1]. Therapeutic monoclonal antibodies (mAb) mediate anti-tumor effects through several distinct mechanisms, including complement-mediated cytotoxicity (CDC), antibody-dependent cell-mediated cytotoxicity (ADCC), and phagocytosis [2]. Thus an important aspect in immunotherapy related outcomes is the availability and activity of the complement (C) cascade. Standard doses of mAb may cause severe reduction of C that depletes the body’s C reservoir and its cytotoxic capacity, and also results in inadequate killing of a second target cell challenge [3]. Thus an important aspect in immunotherapy outcomes is the availability and activity of the C cascade. Extensive evaluation of C components [4] has shown that 70% of the CLL patients had decreased levels of at least one component, in correlation with the stage of the disease. Deficiency of C components, mAb-induced depletion of the C reservoir and the known hypogammaglobulinemia in CLL may increase patients' susceptibility to infections. A recent study that examined C deficiencies in CLL [3] showed a significant proportion of the patients with normal levels of C1q, C2, C3 and C4 but with low C activity, and/or considerable exhaustion of the C system. The data may indicate a role attributed to deficiencies in other C components or to factors other than C concentrations that can affect the overall C activity in these patients. Structural abnormalities of the C components and their association with C function in disease states have not yet been studied.

The classical pathway (CP) is one of the three activation cascades of the C system, which also include the alternative pathway (AP) and the lectin pathway. The CP is triggered by formation of antigen-antibody complexes (immune complexes), assembled from IgM or IgG (in pentamer or hexamer form), which bind the C1 complex (C1q, C1r2, C1s2). Once initiated, the CP proceeds by serial steps of proteolytic activation of the C4 component and formation of the C3-convertase C4b2a which gains C5-convertase activity upon addition of C3b [5,6]. The lectin pathway is activated by the binding of mannan-binding lectin (MBL) or ficolins to carbohydrates and other pathogen-associated molecular patterns that lead to the assembly of the same C3-convertase, C4b2a. The AP is triggered directly by foreign surfaces; for example, by microorganisms or artificial biomaterials. Although these pathways differ in their activating stimuli, they all converge in a common pathway, activating steps of C5b assembly with C6-C9 to form the membrane attack complex (MAC, C5b-9). C5b-9 elicits cell lysis by insertion into the lipid bilayer of the pathogen's cell membranes [6,7].

In this study we show an apparently abnormal form of C5 that has not been described previously in CLL or in other clinical states, and its association with CP activation. The findings are of significant importance for CLL patients receiving immunotherapy treatments.

## Material and methods

All chemicals and antibodies were obtained from Sigma (St. Louis, MO, USA), unless specified otherwise.

### Subjects

Blood samples (10 ml) were collected from 40 naïve CLL patients and 15 healthy normal controls (NC). Plasma and sera were separated immediately and frozen at -70°C. Samples were carefully handled as described [8] in order to avoid spontaneous C activation. Biochemical and hematological parameters, and CLL staging were recorded. The levels of C3 and C4 were quantified using an immunoturbidimetric test (ABBOTT laboratories, USA) on the ARCHITECT clinical chemistry analyzer. The study was approved by the Institutional Review Board of Galilee Medical Center, in compliance with the declaration of Helsinki, and all subjects signed a written informed consent form.

### Analysis of C5 isoforms

Plasma samples were albumin and Ig depleted (commercial kit; Sigma). C3, C4 and C5 were studied under non-reducing conditions by Western blot analysis, using goat polyclonal antibodies (anti-human C3 Santa Cruz Biotechnology; anti-human C4 Quidel) or mouse monoclonal anti-human C5 (Quidel).

### Complement activity

C activation was followed by the levels of soluble C5b-9 (sC5b-9; commercial ELISA, Quidel), and C5a (commercial ELISA, Affymetrix-eBioscience). Activation via the CP or AP was performed in DGVB^2+^ buffer, using aggregated IgG or Zymosan, respectively, as described [9] with a minor modification: serum was diluted to 2.5% in all C activity assays. Changes in CP and AP activities were also followed in sera supplemented with physiological concentration (75μg/ml) of purified C5 [6]. In a separate set of experiments, C activity was studied in C5-deficient serum (Sigma) supplemented with purified normal human C5 (Quidel) or with subjects' serum. The ideal proportion of added subjects' sera for in-vitro activations was established after evaluation of 10%, 20%, 33% or 50% of the sera mixture.

### Assessment of the classical complement convertase

A direct measurement of the convertase level is currently unavailable, however measurement of the convertase activity can be performed [10]. To assess the activity of the CP C5-convertase (C4b2a3b), ethylenediamine tetraacetic acid (EDTA) was added to sera to a final concentration of 30mM, 20mM or 10mM, before CP activation. EDTA acts as a metal chelating agent of ions including Ca^2+^ and Mg^2+^ and inhibits the C cascade at various stages [10,11] by preventing the formation of the intrinsic subject's C5-convertase while preserving the C5 intact. Separately, aggregated IgG was added to C5-deficient serum, activating the CP, which continues only up to the step of the C5-convertase (C4b2a3b) formation and not further, due to the lack of C5. This sample, containing high levels of normal C4b2a3b, was then added to the EDTA-treated serum ("combined" samples), incubated at 37°C and stopped after exactly 10 min as described [9]. C activation was assessed by sC5b-9 levels. Addition of normal C5-convertase to the non-activated subjects' serum containing normal or abnormal C5 enabled the evaluation of the activity of an upstream C complex, independent of C5 structure. Yet, addition of the activated C5-deficient serum to the EDTA-serum caused a dilution of the EDTA-serum, and could potentially enable minute, intrinsic activation of the subject's C system during the 10 min activation of the "combined samples". Also, the preexisting sC5b-9 in the patients' sera could increase the measured sC5b-9 levels, so that both intrinsic activation and preexisting sC5b-9 could potentially influence the results. In order to correct for these interfering factors, intrinsic C activation during 10 min was measured in a separate tube. The sC5b-9 levels measured in these control tubes were subtracted from the final sC5b-9 levels obtained in the "combined" samples. Results were expressed relative to the mean values obtained with NC samples.

### Analysis of C5b in sera

Plasma or serum samples were studied under non-reducing conditions by Western blot analysis using mouse anti-human C5b antibodies (Acris). C5b in sera was also assessed biochemically by the ratio of sC5b-9 to C5a, formed during CP activation. For this purpose, C5-deficient serum was supplemented with mixtures of purified normal human C5 and purified normal human C5b,6 (Fitzgerald Industries International) at molar ratios 3:1; 1:1; 1:3 (0, 25, 50 and 75% C5b,6). CP was then activated, levels of sC5b-9 and C5a were measured, the ratios of sC5b-9 to C5a were calculated and a standard curve was created. The ratios of sC5b-9 to C5a were calculated in the patients' and controls' sera and compared to the standard curve.

### Analysis of Ig-C5a in sera

To assess the possibility that the abnormal C5 form consists of a complex between Ig and C5a, the Ig fraction was precipitated using Sodium sulfate as described [12]. Ig pellets were immediately dissolved in PBS and used for measurement of Ig concentrations (using Nanodrop) and C5a levels (commercial ELISA, RayBiotech). Results were expressed as ng C5a per mg Ig.

### Statistical analysis

Data parameters were analyzed by unpaired t-test, by linear regression analysis and by Wilcoxon Signed Ranks Test, as appropriate. P<0.05 was considered significant.

## Results

### Pattern of complement components

**Fig 1:**
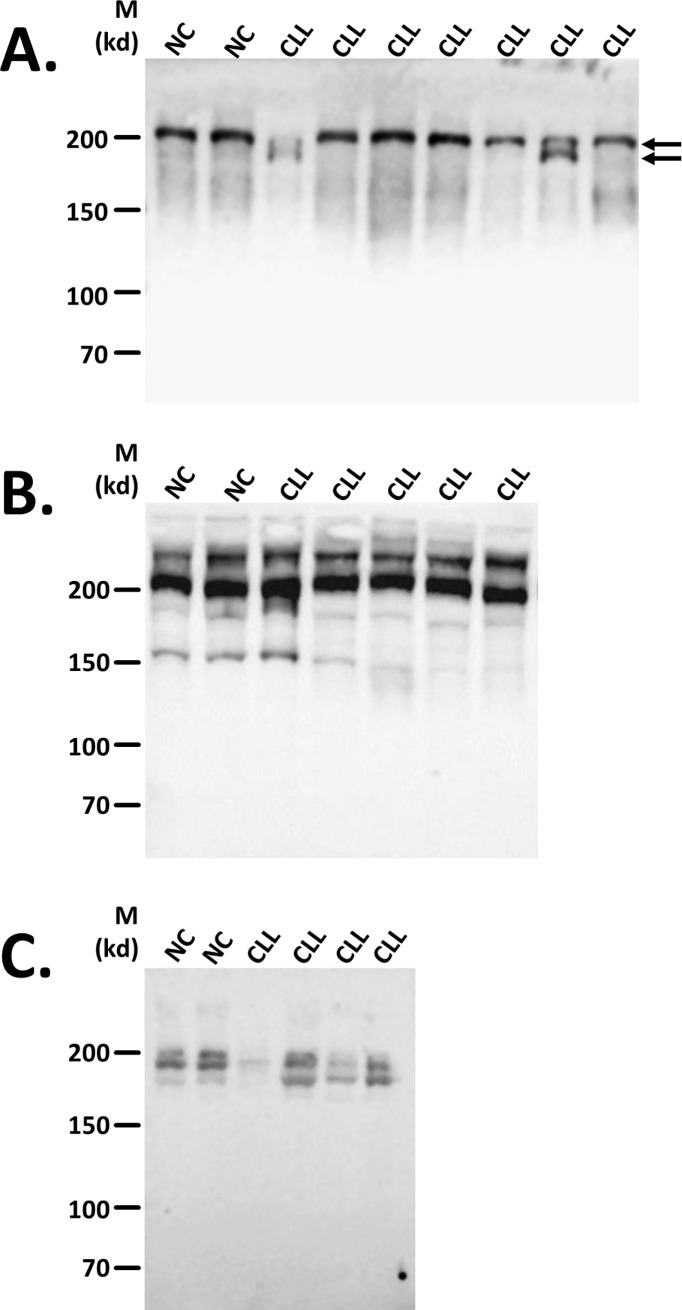
Western blot patterns of complement components. Complement C5 component was followed in plasma under non-reducing conditions, by Western analysis using anti-human C5 (**A**), C4 (**B**) and C3 (**C**) antibodies. The bands of abnormal C5 appearing in some of the CLL patients are indicated by arrows.

C components C3, C4 and C5 were studied in plasma of NC and untreated CLL patients by Western blot analysis, in order to determine structural differences. The C5 component, appearing as a single band in NC, was accompanied in some CLL patients by a lower MW band, forming a double-band and indicating differences in C5 pattern, compared to NC (Fig 1A). This abnormal pattern of C5 was found in 42% of the patients. Western analysis using anti-human C4 and C3 antibodies did not indicate clear pattern differences compared with NC (Fig 1B and 1C).

### Characteristics of the subjects' groups

The characteristics of the NC subjects, CLL patients and the subgroups of patients with normal or abnormal C5 are given in Table 1. C3 levels were found to be significantly higher in CLL patients compared with NC. The majority of patients (>80%) had C3 values within the normal range of 82–185 and 83-193mg/dl for males and females, respectively. Only 15% (6 patients) had values above the normal range and 2.5% (1 patient) were below the normal range (Table 1). When patients were divided according to their C5 appearance, a non-significant difference was observed in the percentage of patients above normal range. The only patient with a low C3 value had normal C5 pattern. C4 levels in CLL patients were similar when compared to NC. The majority of patients (97.5%) had C4 values within the normal range of 15–53 and 15-57mg/dl for males and females, respectively. Only 2.5% (1 patient) had a value below the normal range, with no significant proportional difference between the different groups. Lipid profiles were assessed as an established C activator [13,14]. When comparing the CLL group with abnormal C5 to the other CLL group, no significant differences were found in any of the lipid markers, thus ruling out cholesterol (or other serum lipids) as a confounding factor.

**Table 1:** Characteristics of the study population.

	NC	CLL	CLL	CLL
			normal C5	abnormal C5
**n**	15	40	23	17
**Gender (male/female)**	8/7	26/14	14/9	12/5
**Age (Years)**	53±2*	67±3	69±4	64±6
**Binet Stage (A/B/C)**		28/8/4	7/4/2	11/4/2
**[%A/%B/%C]**		[70/20/10]	[74/17/9]	[65/23/12]
**Serum C3 (mg/dl)**	120±6	151±4^#^	142±7	163±8^#^
Above / Below normal range	0 / 0	6 / 1	2 / 1	4 / 0
% above / % below range	0 / 0	15 / 2.5	8.7 / 4.3	23 / 0
**Serum C4 (mg/ dl)**	28.5±2.1	31.2±1.6	28.5±2.0	34.5±2.2
Above / Below normal range	0 / 0	0 / 1	0 / 1	0 / 0
% above / % below range	0 / 0	0 / 2.5	0 / 4.3	0 / 0
**Beta 2-microglobulin (mg/dl)**	nd	2.3±0.2	2.2±0.3	2.2±0.2
**Serum lipid profile**				
**Cholesterol (mg/dl)**	203±8	166±10	172±12	159±17
**Triglycerides (mg/dl)**	123±26	151±15	139±14	164±27
HDL-Cholesterol (mg/dl)	51±3	32.9±2.0^#^	36.2±2.8^#^	29.2±2.5^#^
Calculated LDL (mg/dl)	136±8	106±8^#^	110±11	101±13
Non-HDL Chol. (mg/dl)	152±8	137±10	138±12	135±17

All values are given as mean ± SEM.

* indicates significant p value (p<0.003) compared to each of the other groups.

# indicates significant p value (p<0.05) compared to the NC group.

### Basal levels of complement activity markers

**Fig 2:**
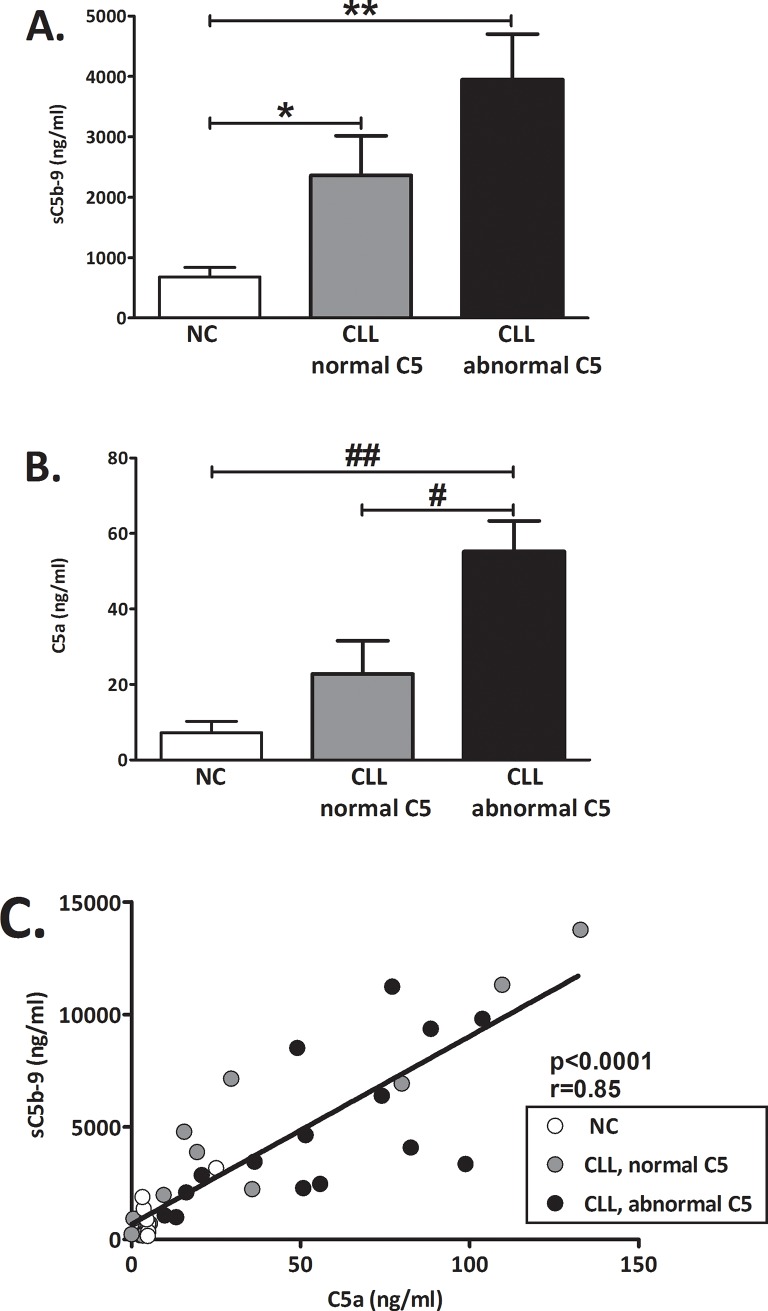
Basal levels of complement activity markers. Basal levels of C activity markers were determined without activation. (**A**) The levels of sC5b-9 were measured in NC (white bars): n = 14; CLL with normal C5 (grey bars): n = 20; CLL with abnormal C5 (black bars): n = 16. *p = 0.043 vs. NC. Values are given as mean with SEM. **p<0.001 vs. NC. (**B**) Basal C activity was also assessed by the levels of C5a in NC: n = 7; CLL with normal C5: n = 14; CLL with abnormal C5: n = 15. ##p = 0.001 vs. NC and #p = 0.012 vs. CLL with normal C5. Values are given as mean with SEM. (**C**) The correlation between the basal levels of C5a and sC5b-9 was analyzed in all subjects. NC (white circles): n = 7; CLL with normal C5 (grey circles): n = 15; CLL with abnormal C5 (black circles): n = 15.

The basal levels (without activation) of the C activity soluble C5b-9 (sC5b-9, the terminal product of the C cascade) and C5a, were measured. Significantly higher basal levels of both markers were found in CLL patients with abnormal C5 pattern (Fig 2). Compared to NC, higher C activity was indicated also in the CLL patients with normal C5 pattern by significantly higher sC5b-9 (p = 0.043, Fig 2A) but not by C5a levels (p = ns, Fig 2B). The levels of the two activity markers were significantly correlated (Fig 2C).

### Complement activation via the CP or AP

**Fig 3:**
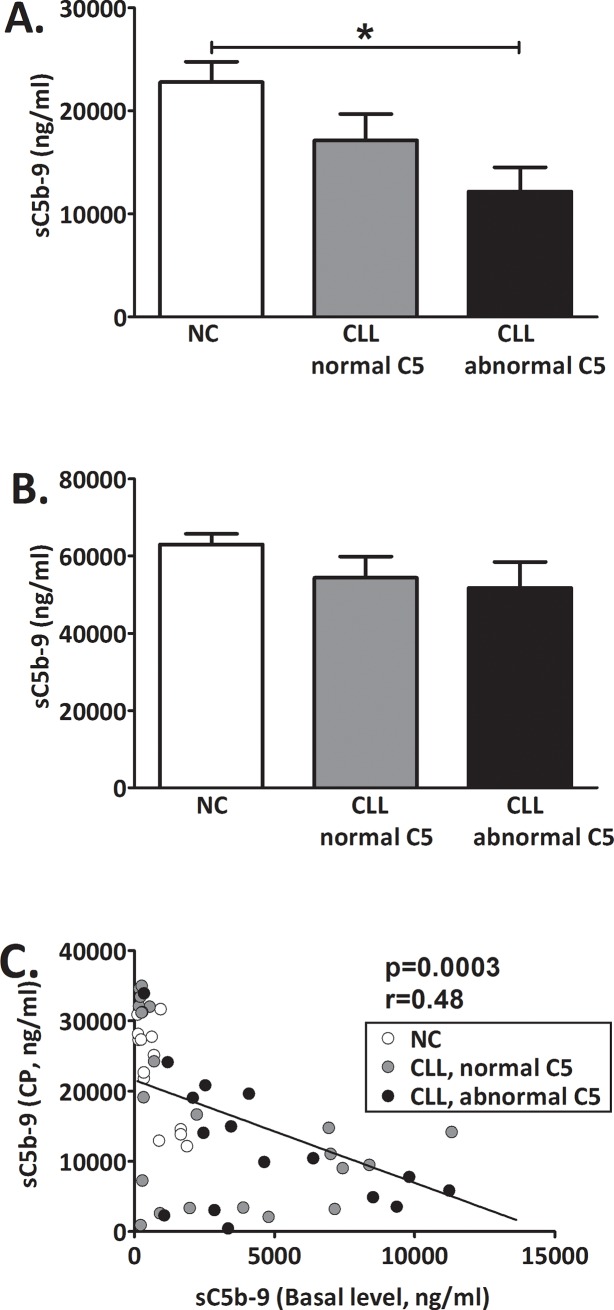
Complement activation in-vitro. C was activated in-vitro via the CP by aggregated IgG (**A**) or via the AP by Zymosan (**B**) and levels of sC5b-9 were determined. Basal sC5b-9 levels were subtracted. Values are given as mean with SEM. NC (white bars): n = 13; CLL with normal C5 (grey bars): n = 23; CLL with abnormal C5 (black bars): n = 16. *p = 0.002 vs. NC. (**C**) The correlation between sC5b-9 levels at baseline and after CP activation was analyzed in all subjects. NC (white circles): n = 13; CLL with normal C5 (grey circles): n = 23; CLL with abnormal C5 (black circles): n = 16.

C activation was induced in-vitro by aggregated IgG or Zymosan that are potent activators of the CP or AP, respectively. When comparing all CLL patients to NC, activation via the CP resulted in sC5b-9 levels that were significantly decreased (p = 0.026). In patients with abnormal C5, sC5b-9 levels after CP activation were significantly decreased by 48% compared to the other subjects' groups (NC and patients with normal C5, Fig 3A). Activation via the AP was comparable in all groups (Fig 3B). The sC5b-9 values measured after CP activation showed a significant inverse correlation with basal sC5b-9 values (Fig 3C).

**Fig 4:**
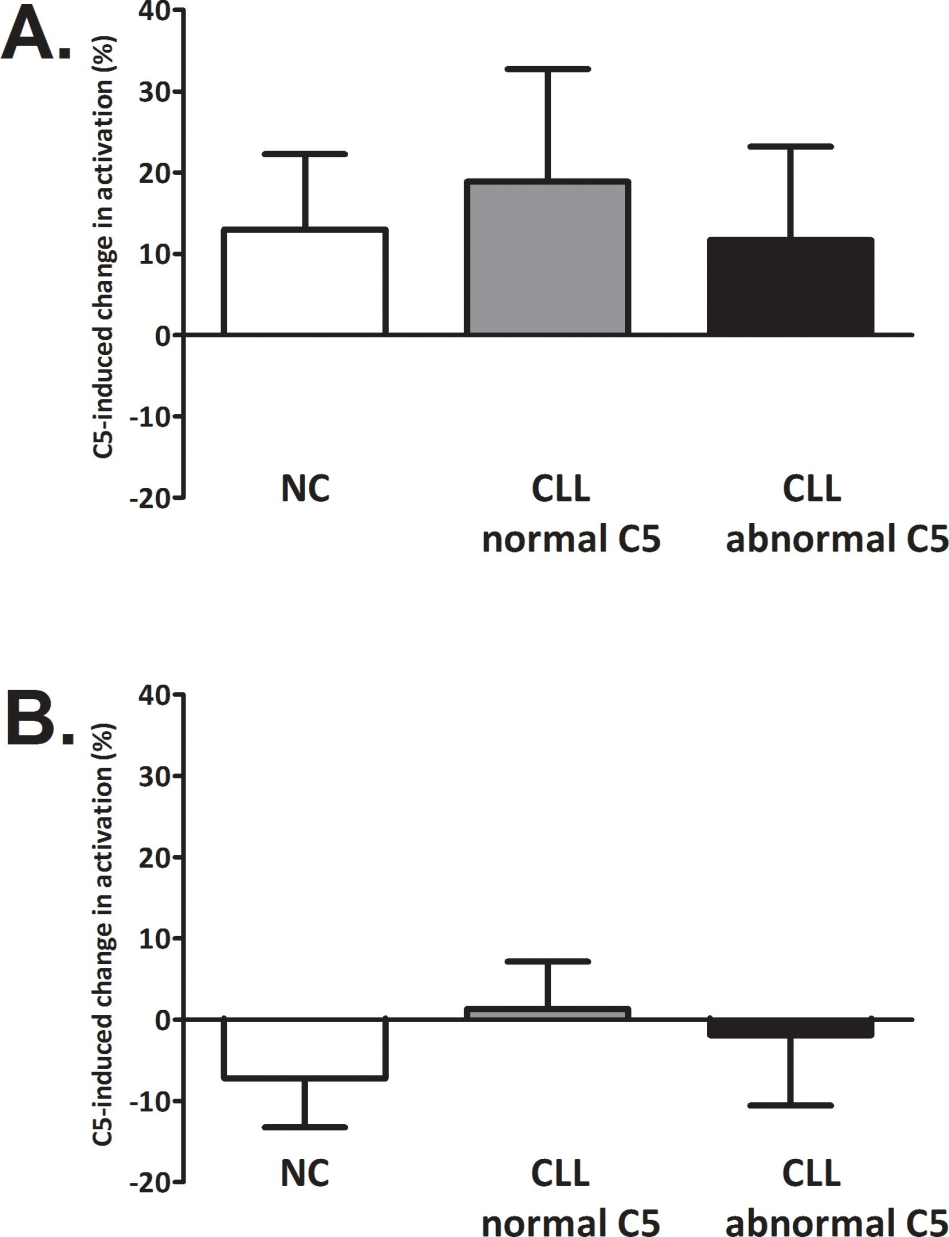
Effects of C5 supplementation on complement activity. Subjects’ sera were supplemented with purified C5 (commercial) to physiological concentration. C activity was assessed by the levels of sC5b-9 after activation with aggregated IgG (classical pathway, **A**) or Zymosan (alternative pathway, **B**). SC5b-9 levels were determined in the sera samples before and after supplementation with C5. The change in sC5b-9 levels due to C5 supplementation is presented as % (relative to non-supplemented sera). Values are given as mean with SD. NC (white bars): n = 9; CLL with normal C5 (grey bars): n = 9; CLL with abnormal C5 (black bars): n = 9.

Although the CP activity appeared to be reduced in CLL patients presenting abnormal C5, it was not clear whether abnormal C5 is responsible for this observation. Moreover, it is unclear whether abnormal C5 is the only irregularity of the C cascade in these patients. In order to answer these questions we conducted two sets of experiments. First, subjects’ sera were supplemented with purified C5 (commercial). The change in C activity due to C5 supplementation (via the CP or AP) is shown in Fig 4. A minor increase in CP activity (12–18%, p≤0.01) ensued C5 supplementation in all groups (Fig 4A), while AP activity was unaffected, though a minute decrease of 7±2% (p = 0.004) was observed in the NC group (Fig 4B).

Altogether, supplementation with C5 had a minute effect on activation in all subjects' groups for both C pathways. More importantly, C5 supplementation did not cause a noticeable increase in activity, suggesting that possible deficiency of normal/active C5 was unlikely.

**Fig 5:**
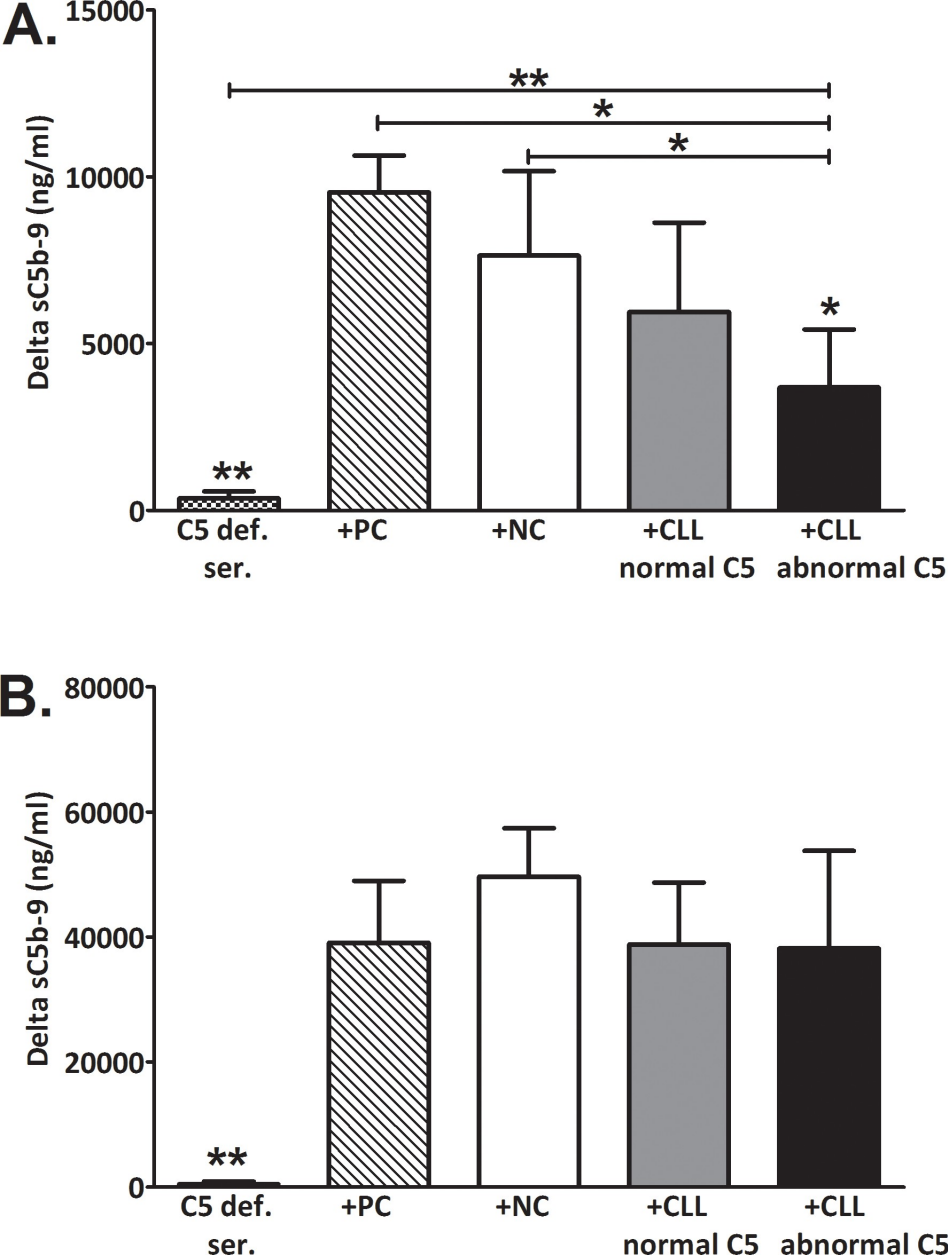
Complement activation in C5-deficient serum supplemented with subjects' sera. C5-deficient serum was supplemented with 33% serum from CLL or NC or with purified C5 (positive control, PC). C activity was assessed by sC5b-9 levels before and after activation with aggregated IgG via the CP (**A**) or with Zymosan via the AP (**B**). The increase (delta) in sC5b-9 levels above baseline is presented (mean with SD). C5-deficient serum before supplementation (squares, n = 3) and in C5-deficient serum supplemented with normal C5 as positive control (PC, Diagonals, n = 3), NC sera (white bars, n = 7), sera from CLL patients with normal C5 (grey bars, n = 6) or sera from CLL patients with abnormal C5 (black bars, n = 7). *p<0.01 vs. all, except for the group of CLL patients with normal C5. **p<0.01 vs. all.

To assess the patients' C5 activity by a more specific approach, another set of experiments was performed, and the activity of a C5-deficient serum was reconstituted by supplementation with CLL or NC serum or with purified C5 (as positive control, Fig 5). The final proportions of 33% subjects' serum and 67% C5-deficient serum were utilized after evaluation of several concentrations, ranging from 10 to 50% (S1 Fig). When the C5-deficient serum was supplemented with 33% subject's serum, the CP activity was significantly lower in patients with abnormal C5, compared with the NC group (Fig 5A). Namely, sera of these patients were unable to reconstitute the CP activity in C5-deficient serum. Activation via the AP was comparable in all groups of subjects (Fig 5B).

### Assessment of the CP C5-convertase

**Fig 6:**
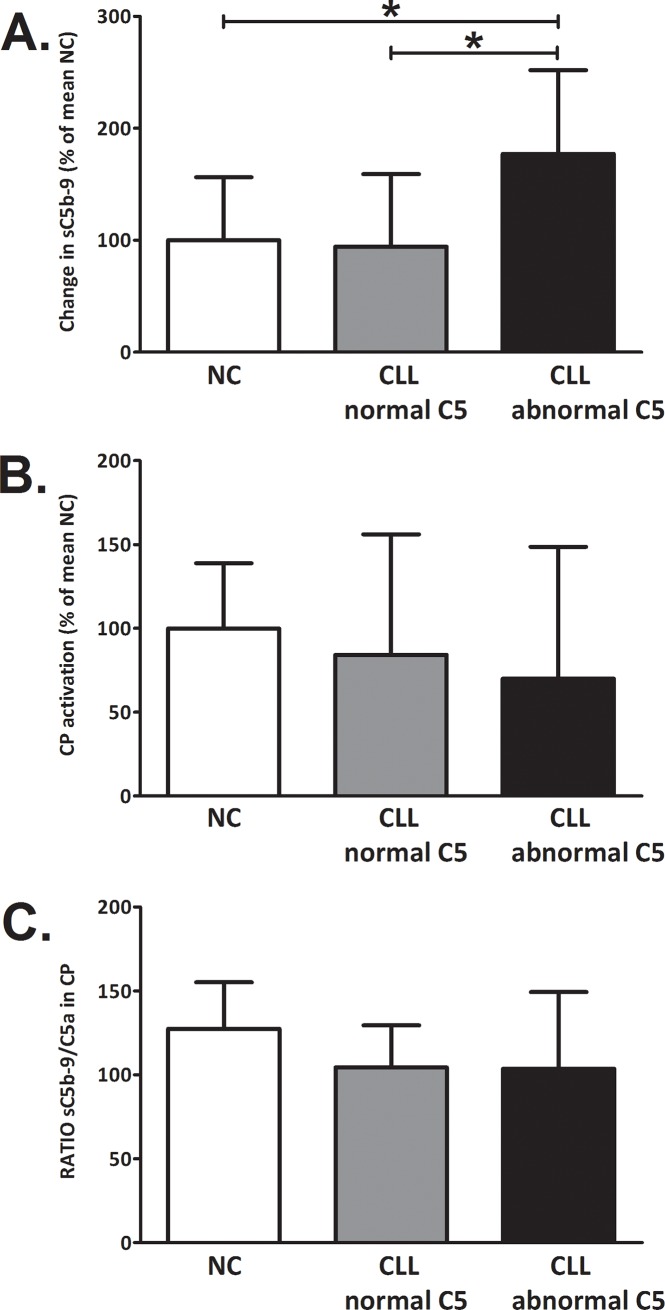
Effects of supplementation with C5-convertase on complement activity. C activation was inhibited in subjects’ sera by EDTA. Sera were then supplemented with classical C5-convertase obtained by IgG-activation of C5-deficient serum. C activity was determined by the levels of sC5b-9 after a short incubation (10 min). The intrinsic C activity of the EDTA-sera, determined in the EDTA-sera prior to supplementation with convertase, was subtracted and the change in sC5b-9 levels was calculates relative to the mean NC value (**A**). Values are given as mean with SD. *p<0.02 vs. NC and vs. CLL patients with normal C5 (Mann-Whitney test). In the same sera samples without EDTA, CP was activated and levels of sC5b-9 (**B**) and C5a were determined. The ratios of sC5b-9 to C5a were calculated (**C**) and values are given as mean with SD. NC (white bars): n = 7; CLL with normal C5 (grey bars): n = 7; CLL with abnormal C5 (black bars): n = 7.

Since C5 supplementation of sera did not increase the activity in patients with abnormal C5, a separate set of experiments was performed to assess whether abnormal C5 is accompanied by additional irregularities of the CP cascade in these patients. C components that act downstream to C5 and are involved in C5b-9 assembly are not likely to be key factors in CLL, because activation via the AP, which involves the same C5-C9 components, was normal in all subjects' groups. Therefore it was assumed that CP components that act upstream to C5 were more likely to be involved in compromising the CP activation. The CP C5-convertase, C4b2a3b, is the immediate upstream complex that can affect CP activity. Its activity was assessed by supplementation of the subjects' serum with normal CP C5-convertase. In patients with abnormal C5, this supplementation caused a significant increase of 77% in CP activity (Fig 6A). Such an increase was not observed in patients with normal C5 (Fig 6A). The observed increase was clearer when using 10mM EDTA (compared to 20mM or 30mM EDTA), (S2 Fig). This increase was due to the supplementation with external convertase and could not be attributed to intrinsic C activation in the samples, which was similar in all the examined sera (Fig 6B). Moreover, in patients with abnormal C5, activation of the CP (without any supplementation) showed a decrease in the production of C5a during CP activation, similar to the decrease in sC5b-9. The constant ratio of sC5b-9 to C5a in all subject groups (Fig 6C) indicated that both C5a and sC5b-9 decreased similarly, supporting the idea that decreased activity of the CP C5 convertase has a role in reducing CP activity in this group of patients.

### Identifying the abnormal C5

**Fig 7:**
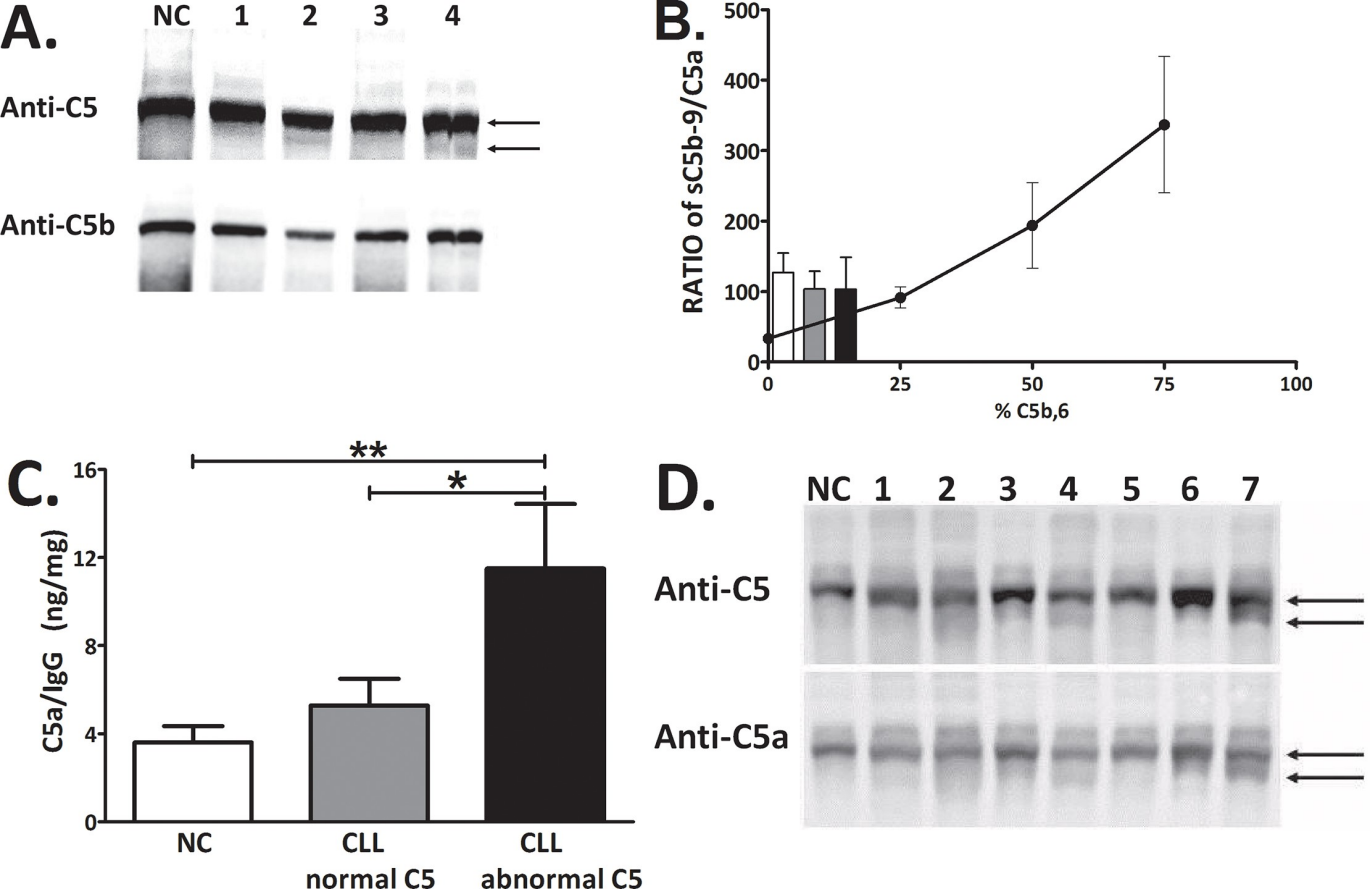
Identifying the abnormal C5. The potential identity of the abnormal C5 as C5b was assessed by Western analysis using anti-C5 and anti-C5b antibodies (**A**). The ratio of sC5b-9 to C5a after CP activation was calculated in subjects' sera and compared to the standard curve of C5-deficient serum supplemented with mixtures of purified C5 and C5b,6 at 0, 25, 50 and 75% C5b,6 (**B**). The potential identity of the abnormal C5 as Ig-C5a complex was studied in Ig fractions that were separated from the subjects' sera and used for measurement of C5a levels (**C**). **p<0.01 vs. NC and *p<0.05 vs. CLL patients with normal C5 (t- test). (**D**) Western analysis of subjects' sera (one NC and 7 patients) using anti-C5 and anti-C5a. The bands of abnormal C5/Ig-C5a complex appearing in some of the CLL patients are indicated by arrows.

Since the C5 component that appeared as a single band in NC, was accompanied in some CLL patients by a lower MW band, forming an abnormal double-band (Fig 1A), we tried to reveal the identity of this abnormal C5 band. We first assessed the possibility that the appearance of a C5 double band could potentially result from activation of the C system and cleavage of the C5 into C5a and C5b. The molecular masses of the proteins (C5:190kDa; C5a:11.2kDa; C5b:180kDa) suggested the possibility that C5 and C5b were identified as a double band. However, both Western and biochemical analyses did not support this possibility. In Western analysis only the normal C5 reacted with anti-C5b, while the lower, abnormal C5 band did not show any signal (Fig 7A). CP activation in subjects' sera resulted in production of sC5b-9 and C5a at a ratio of 115–127 in all subjects' groups (Fig 7B). As the molecular weight of sC5b-9 is estimated to be 1000-1100Da, ~100 fold higher than the 11.2kDa of C5a, the observed ratios of 115–127 essentially represent identical molar concentrations of C5b-9 and C5a, which is expected after activation of sera that contain only C5, and essentially no C5b. Also arguing against C5b as the abnormal C5, is the fact that the ratios found in NC and CLL patients were not statistically different (Fig 7B). Finally, compared with the standard curve, this ratio indicates <20% C5b.

To assess the possibility that the abnormal C5 form consists of a complex between Ig and C5a (Ig-C5a), the Ig fraction was separated and used for measurement of C5a levels (Fig 7C) and for Western analysis (Fig 7D). The levels of C5a measured in the separated Ig fractions were significantly increased in the patients with abnormal C5 (Fig 7C) supporting the presence of an Ig-C5a complex. In agreement, Western analysis using anti-human C5a antibodies indicated a clear signal in the abnormal C5 band (Fig 7D), further supporting the identification of the abnormal C5 as an Ig-C5a complex. Additional confirmation for the presence of an Ig-C5a complex was obtained by immunoprecipitation (IP) experiments, using anti-human C5a antibodies. Using IP the C5a-contatining complex was separated. When the IP complex was used for Western bolt analyses with anti-human IgG antibodies, a clear positive IgG signal was demonstrated, further supporting the formation of an Ig-C5a complex (S3 Fig).

## Discussion

This study describes increased levels of C activity markers and the presence of an Ig-C5a complex, which result from constant in-vivo activation of C in some of the CLL patients. The in-vivo activation probably occurs via the CP, and is associated with decreased ability to activate the CP. In agreement, presence of Ig-C5a is accompanied by a decrease in the activity of the classical C5 convertase.

The serum levels of C components have been studied in CLL patients, showing that in 70% of the patients, the level of at least one of the studied proteins (C1- C9, Factor B and properdin) was decreased [4], in correlation with the stage of the disease. The level of C5 was not decreased in any of the patients described in that study [4]. Structural abnormalities of C components, and particularly C5, were not investigated in that or any other study, to the best of our knowledge. Western analyses in this study indicated an abnormal appearance of C5 and normal patterns of C3 and C4. The in-vivo C activation suggested by the high levels of activation markers could potentially result from cleavage of the C5, which would generate a mixture of C5 and C5b. However, this is not supported by the data. Instead, the formation of a complex between Ig and C5a was indicated. Such a complex has been described previously [15,16] and its assumed role is to quench the harmful effects of activated complement components by preventing them from binding to their specific receptors [16]. This scavenging function of Ig is mediated by low-affinity interaction with the constant domain of the F(ab)’2. The potential spontaneous activation is also supported by the observed increase in baseline levels of the C activation markers, C5a and C5b-9. Direct determination of these C activation markers, indicating in-vivo activation in CLL patients, was not yet reported. Yet, such constitutive C activation in CLL was previously proposed by the exhaustion of C1 and C4 [17,18], associated with decreased hemolytic activity of the CP [18,19]. In this study, the decreased activation via the CP found in CLL patients with abnormal C5, may have resulted from the exhaustion of some CP components, other than C3 and C4 (that were unaffected). Several mechanisms may be involved in spontaneous C activation in CLL patients, including AP activation related to reduced expression of CR1 and CR2 on malignant B cells [20], reduced expression of the C1 inhibitor [18] and the presence of cholesterol crystals [13,14]. With regard to the role of cholesterol, our data indicated no significant differences in the levels of any lipid (cholesterol, triglycerides, HDL, LDL), thus ruling out cholesterol or other major serum lipids as a confounding factor. Altogether, the presence of abnormal C5 (Ig-C5a) was not statistically linked with the measured biochemical markers, or with the CLL staging of the patients.

The study indicated that abnormal C5 (or Ig-C5a) is accompanied by additional C irregularities, as suggested by the inability of normal C5 to restore CP activity and by the results in C5-deficient serum supplemented with patients' sera. The reconstitution of AP, but not CP, activity in C5-deficient serum by sera from patients with Ig-C5a, suggests that the C5 component required for the C cascade was present and active, but other factor/s inhibited the normal CP activation. Supplementation with the immediate upstream complex C4b2a3b, the classical C5-convertase, showed significant increase in activation and supports the possibility that CP C5-convertase activity is compromised in patients with Ig-C5a.

Various mechanisms may compromise this C5-convertase activity in CLL, including decreased C2 levels [3,4]. In these patients, low C2 levels may limit the formation of C2a, a major component of the CP convertases, and thus decrease the classical C5 convertase activity during CP activation. C2a is also a component of the classical C3 convertase, which cleaves the C3 into C3a and C3b. Low concentrations of C2 may therefore affect the levels of C3 in sera, and be related to the increase in serum C3 levels observed in the CLL patients, and especially in patients with abnormal C5. A recent study described the architecture of the CP C5 convertase C4b2a3b and particularly the structure of C4b as a component of this complex [7]. CP C5 convertase differs from CP C3 convertase only by the presence of C3b. Mortensen et al. [5] suggested that C3b shifts the specificity of the convertase complex from C3 to C5 by inducing conformational changes in C4b (rather than a direct interaction with C5). We suggest that in CLL patients, and particularly in patients with Ig-C5a/abnormal C5, the decrease in C2 levels and increase in C3 levels affects the levels of C2a and C3b, can interfere with the balance between available C2a and C3b components of the C5 convertase, and therefore can affect its assembly, levels and activity. Clearly, the factors regulating CP C5 convertase activity in CLL need to be further studied.

The CP activity has great clinical importance in CLL. Low CP activity predicted short survival time in CLL patients [21]. Interestingly, CP activity was the only factor that was significantly related to survival from among the C-related factors investigated, including AP activity, concentrations of C1, C3, C4, factor B and C1-inhibitor [21]. Another important consideration is the role of the CP in CDC, elicited during immunotherapy. The therapeutic approach in CLL commonly includes therapeutic mAbs (immunotherapy), such as Rituximab, Obinutuzumab and Ofatumumab, which target the CD20 expressed on B-CLL cells and mediate their anti-tumor effects through CDC. Thus an important aspect in immunotherapy outcomes is the availability and activity of the C system and particularly the CP that is essential for CDC.

The levels of various C components and activation markers have been measured in both prospective and retrospective studies of various clinical states, showing significant differences between groups of patients. Yet, it is not possible to make predictions for individual patients because of the lack of sensitivity and specificity of many of the assays used. The data presented in this study indicate a link between activation of the C system and appearance of an Ig-C5a complex in CLL, which bears a potential to develop a prognostic tool. It may assist in identifying a sub-group of CLL patients who display abnormal C5 pattern and impaired activity, and are likely to be less responsive to immunotherapy treatment due to compromised CP activity and CDC. This sub-group of CLL patients may benefit from refining and personalizing the immunotherapeutic approach. As part of the personalized approach, CDC may be improved in this subgroup, for example by supplementation with fresh frozen plasma as a source of normal C components, as demonstrated previously [22,23]. Consequently, the study offers a tool for personalizing the immunotherapeutic approach aimed to improve therapy outcomes in CLL.

## Supporting information

S1 Fig Complement activation in C5-deficient serum supplemented with subjects' sera in various proportions. (DOCX)

S2 Fig Assessment of the classical complement convertase. (DOCX)

S3 Fig Immunoprecipitation of the Ig-C5a complex from subjects' sera. (DOCX)
