# Supplementary material for: A C5a-Immunoglobulin complex in chronic lymphocytic leukemia patients is associated with decreased complement activity
Source: PLoS One. 2019 Jan 2;14(1):e0209024. doi: 10.1371/journal.pone.0209024 (PMC6314568; doi:10.1371/journal.pone.0209024)
Supplement: S1 Fig — (DOCX) [file pone.0209024.s001.docx]

**S1**
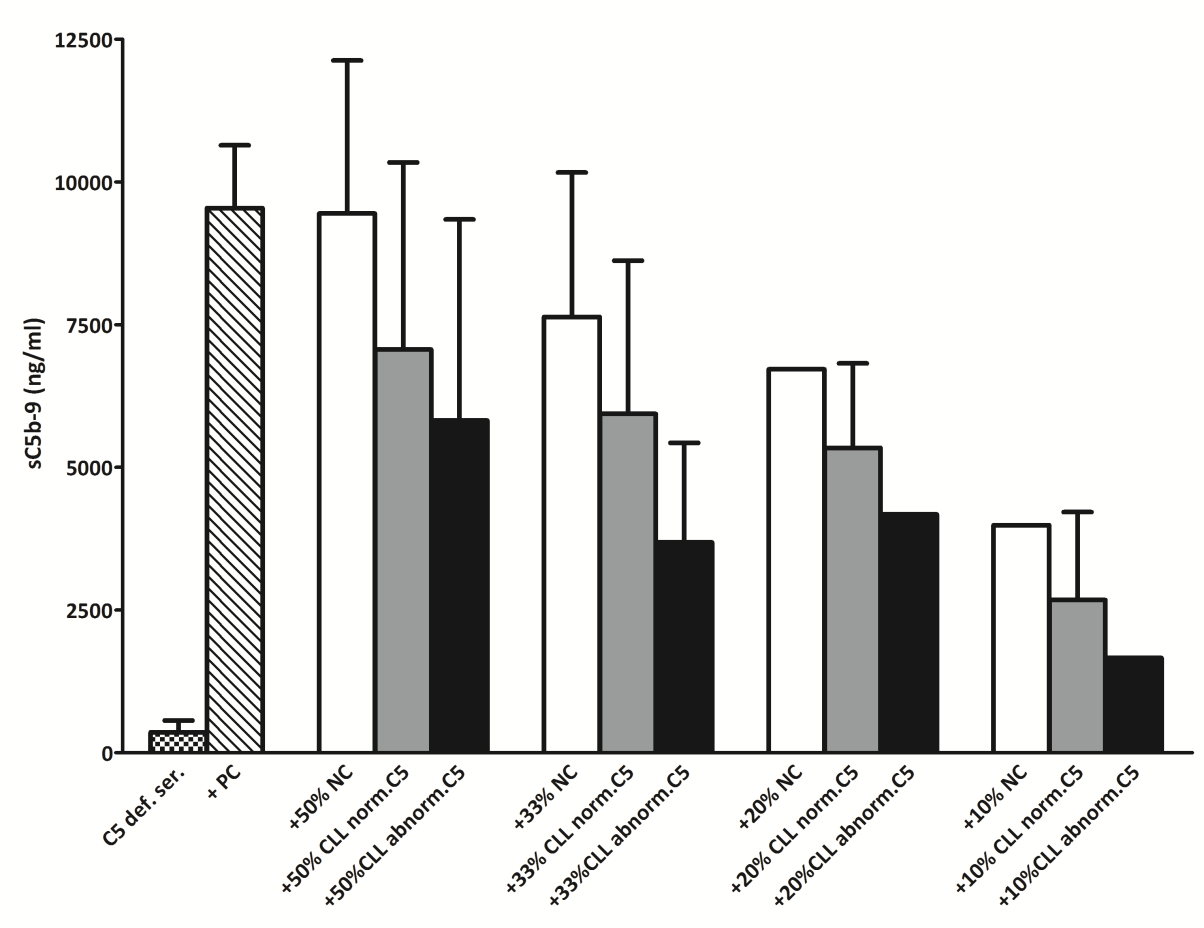
 **Figure**

**S1 Figure.** ***Complement activation in C5-deficient serum supplemented with subjects' sera in various proportions***

In order to assess the activity of the C5 complex from patients in a more specific approach, C5-deficient serum was employed. The proportion of subjects' serum vs. C5-deficient serum in the sera mixture used for the in-vitro activations was determined. C5-deficient serum was supplemented with (+) 10%, 20%, 33% or 50% serum from CLL or HC subjects or with purified C5 (as positive control, PC). C activity was assessed by the levels of sC5b-9 before and after in-vitro activation with aggregated IgG via the classical pathway. The increase (delta) in sC5b-9 levels above baseline is presented (mean with SD). C5-deficient serum before supplementation n=3; C5-deficient serum supplemented with normal C5 as positive control (PC) n=3; C5-deficient serum supplemented with HC sera (white bars): n=7; C5-deficient serum supplemented with sera from CLL patients with normal C5 (grey bars): n=6; C5-deficient serum supplemented with sera from CLL patients with abnormal C5 (black bars): n=7.
