# Supplementary material for: A C5a-Immunoglobulin complex in chronic lymphocytic leukemia patients is associated with decreased complement activity
Source: PLoS One. 2019 Jan 2;14(1):e0209024. doi: 10.1371/journal.pone.0209024 (PMC6314568; doi:10.1371/journal.pone.0209024)
Supplement: S2 Fig — (DOCX) [file pone.0209024.s002.docx]

**S2 Figure**


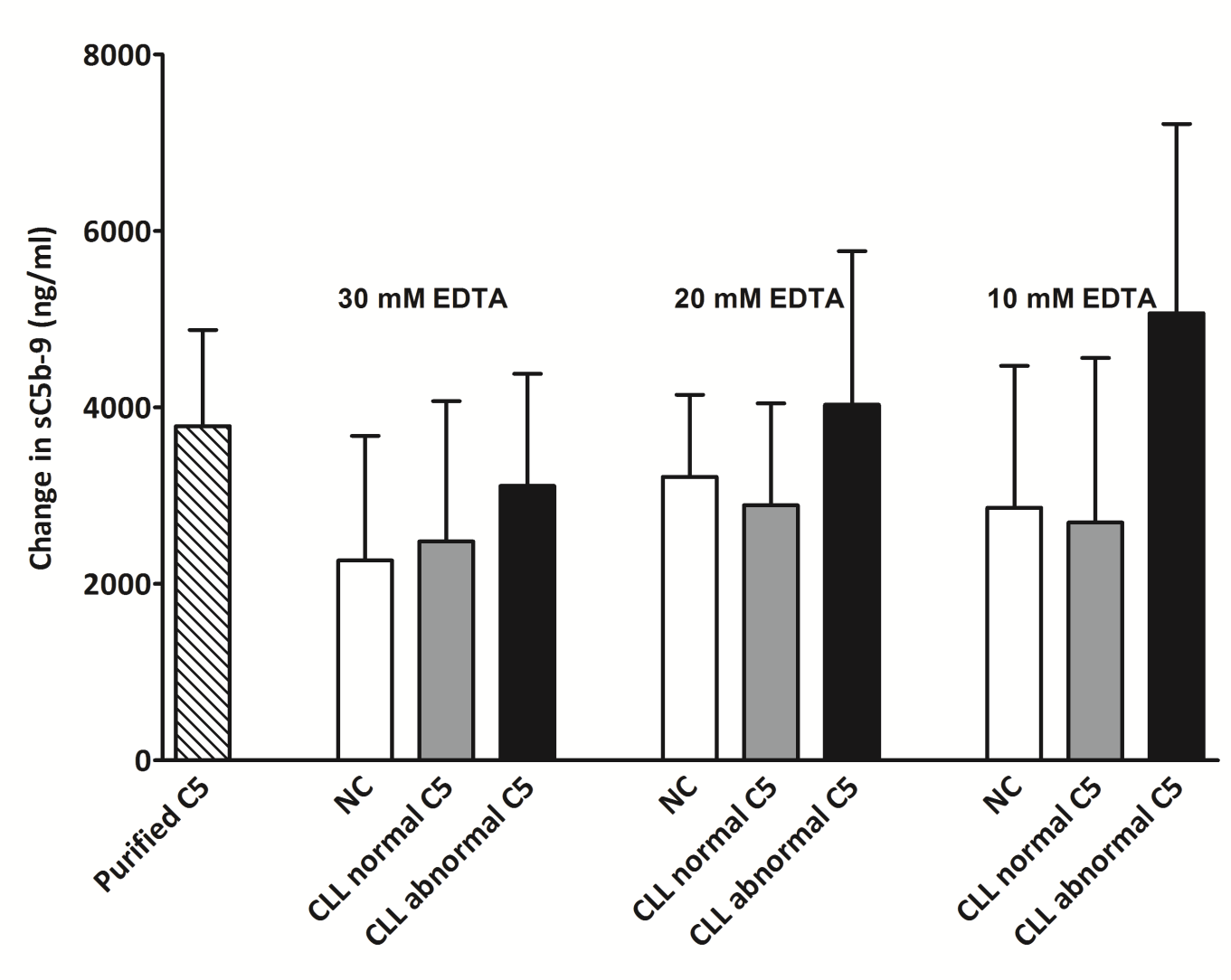


**S2 Figure.** ***Assessment of the classical complement convertase***

C activation was inhibited in subjects’ sera by various concentrations of EDTA. Concentrations of 10mM, 20mM and 30mM EDTA were employed. Sera were then supplemented with classical C5-convertase obtained by IgG-activation of C5-deficient serum. C activity was determined by the levels of sC5b-9 after a short incubation (10 min). The intrinsic C activity of the EDTA-sera, determined in the EDTA-sera without supplementation with convertase, was subtracted and the changes in sC5b-9 levels are presented (mean with SD). *p<0.02 vs. NC and vs. CLL patients with normal C5 (Mann-Whitney test). NC (white bars): n=7; CLL with normal C5 (grey bars): n=7; CLL with abnormal C5 (black bars): n=7.
