# Supplementary material for: A C5a-Immunoglobulin complex in chronic lymphocytic leukemia patients is associated with decreased complement activity
Source: PLoS One. 2019 Jan 2;14(1):e0209024. doi: 10.1371/journal.pone.0209024 (PMC6314568; doi:10.1371/journal.pone.0209024)
Supplement: S3 Fig — (DOCX) [file pone.0209024.s003.docx]

**S3 Figure**


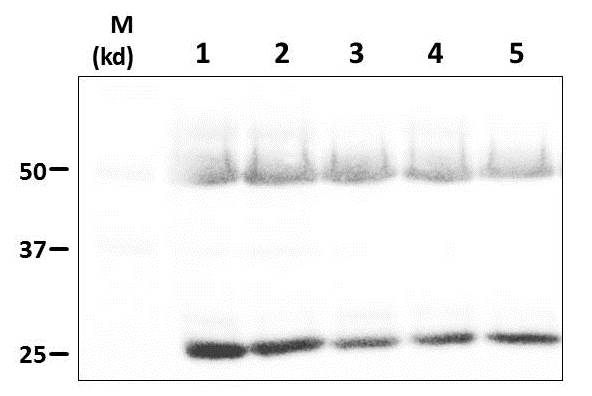


**S3 Figure.** ***Immunoprecipitation of Ig-C5a complex from subjects' sera***

In order to further confirm that the second band identified in the C5 Western blotting is an Ig-C5a complex, immunoprecipitation (IP) experiments were performed. Anti-human C5a antibodies were used for IP of C5a-contatining complexes from sera, which were then analyzed in Western blot for the presence of IgG. A clear positive IgG signal (heavy and light chains) was demonstrated, further supporting the formation of an Ig-C5a complex.
